# Protocol study: sexual and reproductive health knowledge, information-seeking behaviour and attitudes among Saudi women: a questionnaire survey of university students

**DOI:** 10.1186/1742-4755-11-34

**Published:** 2014-05-06

**Authors:** Manal Farih, Khalid Khan, Della Freeth, Catherine Meads

**Affiliations:** 1 Women’s Health Research Unit - Centre for Primary Care and Public Health, Barts and the London School of Medicine and Dentistry, Queen Mary University of London, Yvonne Carter Building, 58 Turner Street, London E1 2AB, UK; 2 Health Economics Research Group, Brunel University, Uxbridge, UK

**Keywords:** Sexual health, Reproductive health, Knowledge, Awareness, Information, University, Students, Saudi Arabia

## Abstract

**Background:**

Sexual and reproductive health (SRH), a basic right for women worldwide, is infrequently researched in countries in the Middle East and North Africa (MENA). No empirical studies of SRH among Saudi women exist. This protocol describes a study to explore the SRH knowledge, information-seeking behaviour and attitudes of Saudi female university students.

**Methods/Design:**

This study will administer a questionnaire survey to female students at 13 universities in Riyadh, Saudi Arabia. The questionnaire was developed following a literature search to identify relevant content, with psychometrically tested tools used when available. The content layout and the wording and order of the questions were designed to minimize the risk of bias. The questionnaire has been translated into Arabic and piloted in preparation for administration to the study sample. Ethical approval for the study has been granted (reference no. QMREC2012/54). After questionnaire administration, the data will be collated, analysed and reported anonymously. The findings will be published in compliance with reporting guidelines for survey research.

**Discussion:**

This study will be the first to provide fundamental information concerning Saudi females university students SRH knowledge and information needs.

## Background

Sexual and reproductive health (SRH) education, a basic right for women, enriches their knowledge and awareness, providing them with the tools to understand their responsibilities and rights [1]. Proper SRH promotion is scarce in many countries, and particularly in developing countries. The disparity between international trends in SRH and the state of affairs in these countries is linked to a shortage of skilled health services and lack of appropriate health services, the shortage of teachers able and willing to communicate about sensitive matter, and religious and cultural taboos [2]. All of these factors may contribute to a diminution in women’s self-confidence and ability to make informed choices. There is limited discussion of issues concerning reproductive health in general, and sexual education in particular, in countries in the Middle East and North Africa (MENA) [3].

Absence of knowledge means that women cannot make or are not in a position to make informed and correct choices, with the consequence that they are likely to suffer from sexually transmitted infections and unwanted pregnancies, which especially in the case of unmarried women can be extremely damaging socially [3].

In Saudi Arabia, women’s SRH is an area, which is usually linked with morality and tradition and very often presented as an aspect of religion. Women’s issues are always linked to Islamic teaching in the Saudi community. Little is known about the knowledge, needs, attitudes or practices of Saudi women in relation to their SRH [4]. The widespread assumption is that women do not need to be knowledgeable about their own SRH, especially if they are single, since it is a taboo topic, which people therefore prefer not to discuss. Moreover, the high social and religious value placed on virginity in most Arab countries, including Saudi Arabia, puts young unmarried women at risk of stigma or negative reactions from health professionals if they try to obtain contraceptive or SRH services or advice [3]. Despite the fact that religious teachings and the prevailing culture in Saudi Arabia prohibit sexual activity or relationships before marriage, such activity may occur. Unfortunately, very little is known about this important aspect of Saudi community, which therefore warrants formal investigation.

A systematic search of the literature on university students’ SRH knowledge and awareness was conducted using electronic databases (MEDLINE, EMBASE, Web of Knowledge, PsycINFO and CINAHL) to identify relevant papers published between January 2012 and January 2013. The reference lists of the selected publications were screened for additional papers of interest, and citation tracking of these papers was used. There were nine questionnaire survey-based studies from five different countries in MENA: Turkey [5-12] Iran [7,8] Egypt [11] Lebanon [13] the United Arab Emirates [9] and Saudi Arabia [10]. There was a general lack of knowledge and limited awareness of SRH matters. The only study on Saudi women [10] focused only on awareness of sexually transmitted diseases and their transmission and prevention. No study was performed in Saudi Arabia concerning SRH knowledge and awareness. To identify Saudi female university students general SRH-related needs, we planned this study to survey their SRH-related knowledge, information-seeking behaviour and attitudes.

## Methods/Design

### Study design

A cross-sectional study will be performed to survey female students from potential participating universities of the 13 universities in Riyadh city using a translated and piloted anonymous questionnaire in 2014 following local approval. Ethical approval has been granted from Queen Mary University of London (reference no. QMREC2012/54).

Initial attempts were made to draw a stratified random sample, but lack of access to complete student lists made such a sample unattainable, so it was decided to use the quota sampling technique for this study.

The use of a quota sampling technique provides more insight into the diversity of the composition of higher education in Saudi Arabia. Thus, the SRH-related knowledge and attitudes of students at private universities may possibly differ from those of students at state universities as a result of social status and class, as private universities usually attract more affluent students. These students may also have access to better resources, such as Internet connectivity on campus, computers and other facilities, which in combination with a greater flexibility on the part of the university administration, may have affected their SRH knowledge and so will influence the research outcomes and results. In addition, quota sampling helped in recruiting students during their daily activities at the university, taking into consideration their busy study schedules. Finally, as the study aimed to compare private with governmental universities and married with single students, this technique helped in recruiting the required sample size from each of these groups.

### Research questions

1. What is the level of SRH knowledge among Saudi female university students?

2. What are the attitudes of Saudi female university students towards sexual relationships and sexual norms?

3. Are there any differences between single and married Saudi female university students regarding SRH knowledge and attitudes?

4. Are there any differences between Saudi female university students at private and state universities regarding SRH knowledge and attitudes?

5. What are the social and individual factors associated with variations in SRH knowledge and attitudes among Saudi female university students, including

1.1 Levels of religiosity and cultural conformity,

1.1 Communication with parents,

1.1 Activity and socialisation and

1.1 Place of residence?

6. What are students’ sources of information and knowledge regarding their SRH?

### Target population

The target population for the purpose of the present study was all Saudi students registered in all of the schools at the participating universities.

### Design of survey questionnaire

The questionnaire was developed following a literature search to identify relevant content, with psychometrically tested tools used when available (see Additional file 1). The study instrument was adapted from a core WHO questionnaire designed by John Cleland, [14] which has been used in several studies related to SRH in many countries. These investigations included a study of norms, attitudes and sexual conduct in Iran [15] and another on the knowledge, attitudes and behaviours of male in the same country [16]. Permission to use this instrument was obtained from the main author of the core survey and the authors of other studies prior to piloting of the questionnaire.

The instrument was modified to produce a self-administered questionnaire that would be consistent with Saudi cultural norms while addressing the research aims and objectives. The content was laid out such that the wording and order of the questions minimised the risk of bias. The survey was translated into Arabic and back translated into English for checking, and the Arabic version was then piloted in preparation for administration to the study sample. In addition, certain changes were made to the original survey to suit the Saudi context in terms of culture, religion, beliefs and politics, but no changes were made to the original scales used for scoring knowledge and attitudes (see Additional file 2).

The questionnaire, covering various aspects of SRH-related knowledge, attitudes and information-seeking behaviour, requires approximately 20 to 30 minutes to complete.

The questionnaire is divided into ten different variables and contains questions related to each variable. The majority of these questions are in the form of close-ended questions, which can be answered easily and quickly, considering the setting of the data collection and the time limits that the students have at the universities. Moreover, this type of question design is easily coded, so the possibility of misclassifying answers can be avoided.

A pilot study was conducted in 2012 on a small sample of students (n = 25) to determine the feasibility of the main study and to identify misinterpretations and items that were frequently missed or that elicited partial responses. After obtaining approval from a state university, the researcher distributed the questionnaires and estimated the time needed for their completion. The researcher then observed whether the students experienced difficulty in understanding the wording of the items. Additionally, the students were asked to give written comments on the length of the questionnaire, its language and any obstacles to their understanding of any items.

It was found that the questionnaire took approximately 20–30 minutes to complete. The participants raised several important issues, in response to which the researcher redesigned the instrument to make the instructions clearer and made several other recommended changes in wording and the terminologies used.

### Access to sample and ethical considerations

Thirteen universities in Riyadh admit female students and each was approached for permission to invite their students to participate in this study. An official letter was issued by the Saudi Cultural Bureau of the Saudi Embassy in London, UK and sent by the researcher to the Ministry of Higher Education in Saudi Arabia, applying for permission for the researcher to gain access to the university sites. A second letter from the Saudi Bureau in London, UK and the study supervisor was issued to facilitate the performance of the research. Attached to all official correspondence were copies of an information sheet describing the research in general, the research aims and objectives and the researcher’s details. No ethical approval was required from the participating universities. As to access however, each university had different rules. Thus, to gain access to each campus and distribute the questionnaire, written or verbal approval had to be obtained from the administrative board of the university in question.

### Sample size

Due to lack of literature about the level of knowledge towards SRH, contraceptive methods and STD’s & HIV among Saudi female university students, it was assumed that 50% would be having low level of knowledge, with a precision of ± 5% (width of 95% confidence interval) at 0.05 level of significance we need to have 384 study subjects With the sensitivity of the questions in the data collection instrument, 20% non-response was considered; hence a total of 460 female university students will constitute the study sample. By considering differences of 15%, 20% and 30% in the risk factors associated with the low level of knowledge of SRH, contraceptive methods and STD’s & HIV in female university students when compared with the good level of knowledge in female university students, at 0.05 level of significance and with 80% power, we need 157, 95 and 40 in each of the two groups of subjects (with low level & good level knowledge). Hence a sample size of 460 will facilitate the descriptive, bivariate and multivariate analysis to achieve the statistical significant estimates [17].

For selection of study subjects, a sampling frame of 13 female universities of Riyadh city will be used. These 13 universities include eight Private and five Governmental sectors. Based on the consent of university authorities, the university female students will approach to participate in the study. Due to sensitivity of the research topic, using random sampling procedures is not feasible; hence a convenient non-random sampling quota method will be used. So a sample of 230 study subjects each from private and Governmental will be obtain.

### Data collection

Data collection will begin in 2014, when the researcher will travel to Saudi Arabia and contact universities in Riyadh. The researcher will then visit the universities to start distributing the questionnaire, based on positive responses to requests for access and approval for the research.

Each participant will first be provided with an information sheet explaining the purpose of the study and stating that the information that they provide will be kept confidential and anonymous. No names or university IDs will be required. Moreover, participants will be assured that they have the right to withdraw from the study at any time or to abstain from responding to any questions with which they are not comfortable or that they find inappropriate. The researcher will be present at all study sites and available to provide verbal information and explanations to the participants. Once initial consent has been obtained from the participants, they will each be given a questionnaire and an envelope in which they will be asked to seal the questionnaire when they are finished to maintain confidentiality. The researcher will recruit students every day between 8:00 am and 4:00 pm in university waiting rooms, canteens and libraries and while students wait for their cars. The researcher will wait for the students to complete the questionnaire and will then collect the sealed envelopes on the same day. The completed questionnaires will be checked at the end of each working day, and all questionnaire data will then be entered into SPSS. Afterwards, all completed and sealed questionnaire forms will be stored in a secure filing cabinet drawer before returning with the researcher to the UK and again being stored in a locked cupboard.

### Data analysis

The data will be analysed using IBM Statistical Package for the Social Sciences (SPSS) software V.20.0. The full data analysis will use t-tests and chi-square tests for bivariate analyses and logistic regression for multivariate analysis. P values <0.05 will be considered as statistically significant.

### Study timeline

Data collection will occur between the ends of 2013–2014. Data analysis will be performed between November and December 2014, with the dissemination of findings occurring shortly afterward.

## Discussion

It is important to remember that this is a small-scale study, so its results will not be generalizable. We acknowledge the limitations of this research, and we will interpret the results cautiously because Saudi Arabia is considered as a conservative country. However, the findings of this study will provide fundamental information concerning Saudi female universities students SRH knowledge and needs. Although significant studies concerning women’s sexual and reproductive needs and problems have been conducted all over the world, there is a dearth of information regarding sexual patterns and reproductive preferences among women in the Middle East in general, and particularly in Saudi Arabia.

Unfortunately, no empirical studies have clarified Saudi women’s SRH needs and knowledge to identify the situation of women’s general SRH in Saudi Arabia. With such broader contextual background, this study will attempt to explore the SRH needs, problems and awareness of Saudi female university students. This study may be considered as unique because no previous studies have directly focused on female university students SRH knowledge and attitudes in Saudi Arabia. This research will also be significant to policy-makers, helping them to enhance SRH services and sexual health education programmes for women in Saudi Arabia.

### Ethical approval

The study has ethical approval from Queen Mary University of London ethical committee (reference no. QMREC2012/54).

## Competing interests

Study components form part of Doctor of Philosophy degree for the author MF. The authors declare that they have no competing interests.

## Authors’ contributions

MF drafted and revised the protocol and manuscript. All authors read and approved the final manuscript.

## Supplementary Material

Additional file 1 Study questionnaire. Click here for file

Additional file 2 Scoring. Click here for file
