# Supplementary material for: Protocol study: sexual and reproductive health knowledge, information-seeking behaviour and attitudes among Saudi women: a questionnaire survey of university students
Source: Reprod Health. 2014 May 6;11:34. doi: 10.1186/1742-4755-11-34 (PMC4113201; doi:10.1186/1742-4755-11-34)
Supplement: Additional file 1 — Study questionnaire. [file 1742-4755-11-34-S1.docx]

# Appendix

## Study questionnaire

**An exploratory study of sexual and reproductive health knowledge, information-seeking behaviour and attitudes among Saudi women: A questionnaire survey of university students.**

**Your correct answers are very important. Please answer each question carefully and honestly.**

**TYPE OF UNIVERSITY**

Private  Government

**Year of study**

Undergraduate  post graduate

**Academic discipline**

1. Medicine
2. Religious studies
3. Science
4. Humanities
5. Arts
6. Other……………………………………….

**SECTION 1: Personal details**

- 1. **How old are you in years?**
  2. **Where do you live?**

North Riyadh

South Riyadh

West Riyadh

East Riyadh

Outside Riyadh

- 1. **What is your current marital status?**

Single

Engaged

Currently married

Widowed

Divorced

Separated

Other (please specify)……………………………..

**1.4 What is your mother’s level of education?**

No education

Primary school

Intermediate school

High school

University graduate

**1.5 What is your father’s level of education?**

No education

Primary school

Intermediate school

High school

University graduate

- 1. **How religious do you consider yourself to be?**

Highly religious

Religious

Somewhat religious

Not at all religious

**1.7 In your views and behaviour, how traditional do you consider yourself to be?**

Highly traditional

Traditional

Somewhat traditional

Not at all traditional

**SECTION 2: Communication with Parents**

**2.1 I feel that I can discuss issues about sex with my mother or father**.

Strongly disagree

Disagree

Agree

Strongly Agree

- 1. **How much did you learn from your mother or father about the following issues?**

**2.2a The ways in which boy’s and girls’ bodies change during puberty?**

Nothing

Some

A lot

**2.3b Menstruation**

Nothing

Some

A lot

**2.4c The sexual and reproductive systems of men and women**

Nothing

Some

A lot

**2.5d Contraception, the means by which one can prevent pregnancy?**

Nothing

Some

A lot

**2.6f Male and female relationships the sexual relationship between man and woman**

Nothing

Some

A lot

**SECTION 3: Activities and socializing**

**3.1 Do you watch satellite TV?**

Yes

No

**3.2 Do you smoke cigarettes?**

Yes

No

**3.3 Do you smoke a sheesha (water pipe)?**

Yes

No

**3.4 Do you drink alcohol?**

Yes

No

**3.5 Do you use illegal drugs?**

Yes

No

**3.6 How often have you had occasion to see photographs or books illustrating sexual activity?**

Never

Once or twice

3 or 4 times

5 times or more

**3.7 How often have you seen films that illustrate sexual activity?**

Never

Once or twice

3 or 4 times

5 times or more

**SECTION 4: Sexual & Reproductive Health Knowledge**

**Indicate whether you think the following statements are true or false, or that you don't know.**

| **Statements** | **True** | **False** | **Don’t know** |
| --- | --- | --- | --- |
| **4.1** A woman can get pregnant the very first time that she has sexual intercourse. |  |  |  |
| **4.2** Condoms are an effective method of protecting against HIV. |  |  |  |
| **4.3** Condoms are an effective method of preventing pregnancy. |  |  |  |
| **4.4** The oral pill is an effective method of preventing pregnancy. |  |  |  |
| **4.5** Women can get pregnant through kissing or touching. |  |  |  |
| **4.6** Withdrawal is an effective method of preventing pregnancy. |  |  |  |
| **4.7** Within the menstrual cycle, there is a period during there is a high possibility of pregnancy |  |  |  |

**4.8** **Have you ever attended any sexual and reproductive health courses as part of compulsory or optional programmes at university?**

Yes

No

**4.9 Before or shortly after you menstruated for the first time did you speak to anybody about menstruation?**

Yes go to 4.10

No go to section 5

**4.10 If yes, to whom did you speak? (Tick all that apply)**

Mother

Father

Sister

Girlfriend

Close female relative (e.g. aunt)

Teacher

Nurse / Doctor

Other (please specify) ………………………….

**SECTION 5: Personal Attitudes towards Sexuality & Gender**

| **Sexual Norms**  How socially acceptable is it these days if: | **Strongly acceptable** | **Acceptable** | **Unacceptable** | **Very Unacceptable** |
| --- | --- | --- | --- | --- |
| 5.1 Unmarried young people socialize with the opposite sex? |  |  |  |  |
| 5.2 Unmarried females have boyfriends? |  |  |  |  |
| 5.3 Unmarried females have physical intimacy such as touching, kissing and hugging the opposite sex? |  |  |  |  |
| 5.4 Unmarried females have sex? |  |  |  |  |
| **Personal attitudes**  Young people have various views about relationships. What is your personal opinion of the following statements? | **Strongly agree** | **Agree** | **Disagree** | **Strongly disagree** |
| 5.5 It is acceptable for young people to socialize with the opposite sex. |  |  |  |  |
| 5.6 It is all right for unmarried boys to have girlfriends. |  |  |  |  |
| 5.7 It is all right for unmarried girls to have boyfriends. |  |  |  |  |
| 5.8 It is not all right for boys and girls to have physical intimacy, such as touching, kissing and hugging the opposite sex. |  |  |  |  |
| 5.9 It is wrong for unmarried boys and girls to have sexual intercourse, even if they love each other. |  |  |  |  |
| 5.10 Girls should remain virgins until they marry. |  |  |  |  |
| 5.11 It is all right for boys and girls to have sex before marriage if they use methods to prevent pregnancy. |  |  |  |  |
| 5.12 Virginity is a girl’s most valuable possession. |  |  |  |  |
| 5.13 My religious and beliefs are against premarital sex. |  |  |  |  |
| 5.14 It is okay for boys to have sex before marriage but not so for girls. |  |  |  |  |

**SECTION 6: Contraception knowledge and usage**

**6.1 Which methods of contraception have you heard of or know?**

| **Methods** | **Yes** | **No** |
| --- | --- | --- |
| Female sterilization |  |  |
| Pill |  |  |
| Intrauterine device (IUD) |  |  |
| Implant |  |  |
| Lactation amenorrhea method |  |  |
| Injection |  |  |
| Condom |  |  |
| Male sterilization (Vasectomy) |  |  |
| Diaphragm, Foam, Jelly, Suppository |  |  |
| Rhythm or Calendar Method |  |  |
| Withdrawal |  |  |

**6.2 If applicable, do you use any contraceptive methods now?**

Yes Go to question 6.4

No go to the next question

**6.3 If No, why you are not using any method of contraception? (Tick all that apply)**

Not married

Fertility related reason

Opposition to use

Lack of knowledge, knows no method

Religious reasons

Fear of side effects

Husband refuses

Money problems

Lack of access

Other reasons (please specify).......................... Go to section 7

**6.4 What method of contraception are you using? (Tick all that apply)**

Female sterilization (Tubal ligation)

Male sterilization (Vasectomy)

Pill

Intrauterine device

Injection

Implants

Condom

Diaphragm, Foam, Jelly, Suppository

Lactation amenorrhea method

Rhythm or calendar method

Withdrawal

Other (please specify).....................................

**6.5 If applicable, from where did you obtain supplies of the contraception you selected in question 6.4? (Tick all that apply)**

Governmental Hospital

Primary health care centre

Private hospital/ clinic

Pharmacy

Other (please specify).......................................

**6.6 Where did you obtain information on the contraceptive method you are using? (Tick all that apply)**

Doctor

Friends

Family members

My mother

Internet

Husband

Other (please specify).......................................

**Section 7: Sexually transmitted diseases and HIV knowledge**

**7.1 Which sexually transmitted diseases (STD) have you heard of or know?**

| **STD** | **YES** | **NO** |
| --- | --- | --- |
| Gonorrhoea |  |  |
| Syphilis |  |  |
| Chlamydia |  |  |
| HIV/AIDS |  |  |
| Genital herpes / sore |  |  |
| Don’t know any |  |  |

**7.2 Where did you hear about any of these sexually transmitted diseases and HIV? (Tick all that apply)**

Radio

TV

Newspapers

Poster

Health Professional

Mosque

School

Friends

Clinic

Other (please specify)........................................

**7.3 Which symptoms do you think someone with a sexually transmitted disease may have? (Tick all that apply)**

No symptoms

Abdominal pain

Genital itching

Redness in the genitals

Genital lesions/sores

Pain

Burning sensation during urination

Discharge from penis

Vaginal discharge

Weight loss

Infertility

Don’t know

Other (please specify)......................................

**7.4 How do you think one can be infected with a sexually transmitted disease and HIV? (Tick all that apply)**

Sexual intercourse with sex worker

Sexual intercourse with many partners

Not using condom during intercourse

Blood transfusion

Sharing needles

Kissing

Using public toilet

Other (please specify)..................................

**7.5 Is there anything that a person can do in order to avoid sexually transmitted diseases and HIV?**

Yes

No

Don’t know

**7.6 Can a person get the HIV virus from mosquito bites?**

Yes

No

Don’t know

**7.7 Could shaking hands or hugging transmit HIV?**

Yes

No

Don’t know

**7.8 Can people get the HIV virus by sharing food with a person who has HIV?**

Yes

No

Don’t know

**7.9 Do you think HIV is curable?**

Yes

No

Don’t know

**SECTION 8: Information-seeking behaviour and needs**

**8.1 Have you ever talked with anybody about sexual and reproductive health matters?**

Yes

No

**8.2 Who are the people you most often talk with about sexual and reproductive health matters? (Tick all that apply)**

Mother

Father

Sister/brother

Spouse

Teacher

Religious teacher

Friends

Other relatives

Physician

Pharmacist

Nurse

Other (please specify) ………..........................

**8.3 Do you think it is easy to obtain information on sexual and reproductive health?**

Yes Go to question 8.5

No Go to the next question

**8.4 If No, why is it not easy?**

Don’t know where to obtain information

Parents disapprove

No services available

Services providers disapprove

I feel shy

Other (please specify)..................... Go to next question

**8.5 If you had a problem or questions about sexual and reproductive health, where would you go for help? (Tick all that apply)**

Clinic/hospital

Qualified doctor

Spouse

Parents

Traditional healer

Friends

Teachers

Other (please specify)..............................

**8.6 Would you like to attend any courses on sexual and reproductive health?**

Yes

No

**8.7 Do you think sexual and reproductive health education would increase the incidence of sex practices?**

Yes

No

**8.8 In your opinion, which of the reproductive and sexual health services listed below should be provided to women?**

| **Service** | **Yes** | **No** |
| --- | --- | --- |
| Information or classes on reproductive and sexual health matters. |  |  |
| Clinics for sexual and reproductive health problems. |  |  |
| Contraceptives and instruction for use. |  |  |
| Treatment and information on sexually transmitted diseases. |  |  |
| Ante-natal and post -natal classes. |  |  |
| Classes on sexual relationships and premarital preparation |  |  |

**8.9 Is there anything that you want to add?**

**......................................................................................................................................................................................................................................................................................................................................................................................................................................**

**8.10 Do you use the internet?**

Yes

No

**8.11 What kind of information on the internet would you want to know regarding your sexual and reproductive health? (Tick all that apply)**

Sexual intercourse

Genital hygiene

Contraception

Pregnancy and delivery

Sexual problems

Sexually transmitted diseases

Virginity

Other, please write what you information you want to know...........................................

**8.12 Do you consider the information on the internet to be reliable?**

Reliable

Unreliable

Uncertain

**SECTION 9: (If you are married, please complete this section. If not, go to section 10)**

**9.1 How old were you when you got married?**

Age in years

**9.2 Have you had any experience of an unwanted pregnancy?**

Yes

No

**9.3 If applicable, how old were you when you get pregnant for the first time?**

Age in years

**9.4 Have you ever experienced any sexual or reproductive health problems/disease?**

Yes go to the next question

No go to question 9.6

**9.5 If yes, can you tell me what kinds of problems you have had?**

Sexually transmitted disease/ infection

Urinary tract infection

Premenstrual tension

Vaginal itching and burning

Problem with menstruation

Excessive vaginal discharges

Pain during intercourse

Low sexual desire

Fertility problems

Other (please please write any problems you had)............................

**9.6 Have you ever needed to seek medical care or advice for these concerns or needed any help or advice regards sexual and reproductive health matters?**

Yes Go to next question

No Go to page 18

**9.7 If yes, was it helpful?**

Yes

No

**9.8 Where did you go?**

Governmental hospital

Private clinic /hospital

Friends

Other (please specify).............................

**SECTION 10: If you are NOT married please complete this section**

**10.1 Have you ever had any sexual or reproductive health problems or concerns?**

Yes Go to the next question

No Go to question 10.3

**10.2 If yes, what kind of problems or concerns did you have?**

How my genitalia look

Urinary tract infection

Premenstrual tension (PMS)

Hygiene

Excessive vaginal discharges

What is the hymen?

Will I lose my virginity if I play a sport or ride a horse?

Other (please write any problems you had)...................... Go to question 10.3

**10.3 Have you EVER needed to seek medical care or advice for these concerns or needed any help or advice regard sexual and reproductive health matters?**

Yes Go to the next question

No

**10.4 If yes, was it helpful?**

Yes

No

**10.5 Where did you go?**

Governmental hospital

Private clinic /hospital

Friends

Other (please specify)...................................

**Dear students**

**Please place the questioner in the envelope provided then seal it after that return the sealed envelope to the researcher.**

**THANK YOU VERY MUCH FOR COMPLETING THE QUESTIONNAIRE. YOUR HELP IS VERY MUCH APPRECIATED.**

If you have any suggestions or other concerns in this regard please feel free to state them here.
