# Supplementary material for: Protocol study: sexual and reproductive health knowledge, information-seeking behaviour and attitudes among Saudi women: a questionnaire survey of university students
Source: Reprod Health. 2014 May 6;11:34. doi: 10.1186/1742-4755-11-34 (PMC4113201; doi:10.1186/1742-4755-11-34)
Supplement: Additional file 2 — Scoring. [file 1742-4755-11-34-S2.docx]

# Appendix .2

# Scoring

***SRH knowledge*** was assessed by asking the students to identify the accuracy of seven statements. Each correct answer scored 1, each incorrect answer or ‘don’t know’ scored 0. Thus the total scores ranged from 0-7.

***Knowledge of contraceptive methods*** was assessed through awareness of eleven different methods. A score of 1 was given for each method known, giving a total score range of 0-11.

***Knowledge about STDs and HIV:*** Students were asked several questions on STDs and HIV, such as whether they had heard of any of the five STDs listed, modes of transmission, protection from infection, whether HIV is curable and the different signs and symptoms of STDs. A summary index

That assigned a score of 1 for each correct answer and score of 0 for each incorrect or ‘don’t know’ answer gave a total score in the range of 0-25.

***Attitudes toward premarital sex*:** Respondents were asked to assess the social acceptability in the community of certain sexual attitudes, by choosing among four statements, ranging from ‘very acceptable’ to ‘very unacceptable’. Their personal attitudes to some sexual norms and attitudes were tested by their responses to ten statements, ranging from ‘completely agree’ to ‘completely disagree’. These responses were converted to scores on four-point Likert scales, ranging from 1 for extremely conservative views to 4 for extremely liberal views.
